# Research progress on the role of ferroptosis in cardiovascular disease

**DOI:** 10.3389/fcvm.2022.1077332

**Published:** 2022-12-22

**Authors:** Han Li, Li Lin, Yun-Long Xia, Yunpeng Xie, Xiaolei Yang

**Affiliations:** ^1^Institute of Cardiovascular Diseases, The First Affiliated Hospital of Dalian Medical University, Dalian, China; ^2^Department of Cardiology, The First Affiliated Hospital of Dalian Medical University, Dalian, China

**Keywords:** ferroptosis, cardiovascular disease, iron overload, lipid peroxidation, arrhythmia

## Abstract

The cardiovascular disease pathogenesis is extremely complex and seriously threatens human health. Cardiomyocyte death plays a significant role in cardiovascular disease occurrence and development. In addition to the previously revealed modes of cell death (apoptosis, autophagy, and pyroptosis), ferroptosis is highly related to the development of cardiovascular diseases, including arrhythmia, atherosclerosis, and myocardial ischemia/reperfusion. Ferroptosis is a novel cell death pathway driven by lipid peroxidation and iron overload. Lipid, amino acid, and iron metabolism regulate the ferroptosis pathway. Small molecule compounds (iron chelators, antioxidants, and ferroptosis inhibitors) and genetic programming can alleviate or prevent cardiovascular disease by inhibiting the ferroptosis pathway. Ferroptosis plays a key role in various cardiovascular disease occurrence and development, and inhibiting ferroptosis in cardiomyocytes is expected to become a feasible treatment method. In this mini-review, we systematically summarize the molecular mechanisms of ferroptosis in different cardiovascular diseases, delineate the regulatory network between ferroptosis and cardiovascular diseases, and highlight its potential therapeutic targets.

## 1 Introduction

Obesity, hypertension, a high-cholesterol diet, and other factors contribute to cardiovascular disease, endangering human physical and mental health (1). Recently, cardiovascular disease has been associated with multiple cell death pathways, such as ferroptosis, pyroptosis, and autophagy (2). Ferroptosis was officially identified as a novel mode of regulating cell death in 2012, attracting attention to the study of cardiovascular disease (3). Ferroptosis is mainly caused by iron-dependent cell death, accumulating lipid peroxidation to lethal levels, resulting in cell membrane damage (4). The ferroptosis mechanism might result from glutathione depletion, excess iron, and reactive oxygen species (ROS) overgeneration (5). This mini-review mainly describes ferroptosis metabolic pathways and their strong correlation with cardiovascular disease.

## 2 Molecular and metabolic mechanisms of ferroptosis

### 2.1 Lipid oxidation metabolism

In cell membranes, phospholipid-related polyunsaturated fatty acids (PUFAs), such as phosphatidylethanolamine (PE) and phosphatidylcholine (PC), are responsible for inducing lipid peroxidation to induce ferroptosis (6, 7). Acyl-CoA synthetase long-chain 4 (ACSL4) and lysophosphatidylcholine acyltransferase 3 (LPCAT3) are two important enzymes in lipid metabolism (5). These two enzymes can activate PUFAs, such as arachidonic acid (AA) and adrenic acid (ADA), to generate corresponding PE-AA and PE-ADA, respectively (8). Subsequently, lipoxygenase (LOX) can oxidize them to PE-AA-OOH and PE-AdA-OOH to promote ferroptosis product synthesis (8). The glutathione peroxidase 4 (GPX4) transforms toxic lipid hydroperoxides into non-toxic alcohols. Nevertheless, inhibiting GPX4 enzymatic activity causes significant lipid hydroperoxide accumulation, leading to ferroptosis (8).

### 2.2 Glutamate metabolism

A heterodimer containing a light chain SLC3A2 (4F2hc) and a heavy chain SLC7A11 (xCT) is known as cystine/glutamate antiporter (System Xc^–^) on the cell membrane that promotes the glutathione (GSH) synthesis by exchanging extracellular cystine with intracellular glutamate (9). Erastin, an authoritative System Xc^–^ inhibitor, inhibits cysteine absorption to the cellular membrane, leading to glutathione depletion (10). GSH can reduce ROS and reactive nitrogen under the activity of GPX4. Furthermore, GPX4 converts GSH into glutathione disulfide (GSSH) in the oxidation reaction, expunges excessive peroxides and hydroxyl radicals during cell metabolism, and alleviates the PUFAs peroxidation (11). The GPX4 inhibition, like Ras-selective Lethal small molecule 3 (RSL3), causes ferroptosis by activating lipid peroxidation (10). Herein, the System Xc^–^-GSH-GPX4 axis system plays a significant position during ferroptosis.

### 2.3 Iron metabolism

Iron absorption, transport, storage, and excretion affect iron homeostasis, which is crucial for human health (5). Transferrin receptor 1 (TFR1) recognizes ferric (Fe^3+^) by binding to transferrin through the cellular membrane, and iron reductase reduces it to ferrous (Fe^2+^) (12). Then, Fe^2+^ is transferred into the cytosolic labile iron pool (LIP) through divalent metal transporter 1 (DMT1) (5). Moreover, a part of iron is stored in the ferritin as Fe^3+^, while another part is released into the extracellular by membrane transporters ferroportin (FPN) (5).

Iron homeostasis is important for cellular metabolism, especially for cardiomyocytes with high energy requirements (13). Iron overload is a consequence of iron intake exceeds the capacity of transferrin iron-binding, leading to the accumulation of iron in the parenchymal cells of various tissues and organs (14, 15). Ferroptosis is dependent on iron-dependent lipid peroxidation, which can be inhibted by iron chelators (14, 16). Cellular labile iron contents may affect the ferroptosis sensitivity (17). Overflowing Fe^2+^ catalyzes the hydroxyl and high ROS generation through the Fenton reaction, inducing lipid peroxidation and cell damage (5, 11). Heme oxygenase-1 (HO-1) also affects ferroptosis sensitivity by degrading heme to release iron (17).

### 2.4 Other related signaling metabolism

The redox-sensitive transcription factor, nuclear factor erythroid-2 related factor 2 (NRF2) regulates its downstream target genes to modulate lipid peroxidation and ferroptosis (18, 19). NRF2 targets, such as FPN and ferritin, are involved in iron/heme metabolism, which is crucial for cellular antioxidant defense (20). Besides, NRF2 plays a critical role in mediating glutamate metabolism targets, SLC7A11 and GPX4 (19). Of note, NRF2 target protein NAD(P)H:quinone oxidoreductase 1 (NQO1) also plays a key role in regulating ferroptosis (21).

## 3 Ferroptosis in cardiovascular disease

### 3.1 Arrhythmia

Atrial fibrillation (AF) is the most clinically diagnosed arrhythmia (22). Most patients with AF are prone to recurrent attacks, presenting clinicians with a dilemma (23). The mechanical function and electrical activity are progressive deterioration in iron-overloaded hearts. Iron overload can affect calcium, sodium, and potassium channels, interfering with cardiac electrophysiology and confirming the connection between iron ions and arrhythmia (24).

Recently, ferroptosis has gained increased attention in the field of arrhythmia. Antioxidant factor NRF2 overexpression can reduce the arrhythmia, inflammation, and cardiac fibrosis induced by AF (23). Down-regulation the expression of FPN, a downstream factor of NRF2, causes intracellular iron accumulation in the new-onset AF model, leading to ferroptosis (25). Moreover, excessive alcohol consumption can lead to iron dysregulation, increased serum non-heme iron concentration, and atrial tissue iron accumulation. This progress promotes ferroptosis and increases the susceptibility to AF. Ferrostatin 1 (Fer-1) can partially or completely reverse the atrial damage caused by excessive alcohol intake (26).

Arrhythmia is a common clinical feature of coronavirus disease 2019 (COVID-19) (27). According to a recent study, severe acute respiratory syndrome coronavirus 2 infections derange human sinoatrial node-like pacemaker cells, facilitating ferroptosis. Early deferoxamine and imatinib administration reduces viral infection in human embryonic stem cell cardiomyocytes. This result suggests that ferroptosis is involved in the arrhythmia progression in COVID-19 patients (28).

### 3.2 Myocardial ischemia-reperfusion injury

Myocardial ischemia-reperfusion injury (IRI) is tissue injury due to the recovery of blood supply in myocardial tissue, leading to life-threatening clinical complications after a period of ischemia (12). Iron and lipid metabolism are strongly linked to the pathological process of IRI. In the early stage of ischemia and reperfusion, ferritin degradation releases iron, promotes free iron-mediated Fenton reaction and induces oxidative damage (29). Cardiomyocytes are more vulnerable to injury than endothelial cells during the ischemic phase (30). The early ischemic stage, a disorder of PUFAs-phospholipids, may initiate peroxidative conditions, providing a priming signal for oxidative injury in the reperfusion stage. Studies have found that oxidative phosphorylation of the core enzyme ALOX15 can initiate PUFA-phospholipid peroxidation and enhance the susceptibility to ferroptosis in ischemia-induced myocardial injury (31). Consequently, inhibiting ferroptosis during the early stage of ischemia can reduce myocardial injury caused by reperfusion as soon as possible.

Ferroptosis can also regulate IRI through other metabolic pathways. A novel long non-coding RNA LNCAABR07025387.1 is up-regulated in myocardial tissue of IRI rat models, efficiently activates ACSL4 expression by down-regulating miR-205, accelerates the lipid peroxidation and exacerbates IRI (32). Moreover, ROS is strongly related to endoplasmic reticulum (ER) stress during ferroptosis. In diabetic rats during IRI, ER stress factor expressions, such as activating transcription factor 4 (ATF4), C/EBP homologous protein (CHOP), and ACSL4, are elevated. Meanwhile, the GPX4 level is decreased, exacerbating myocardial injury (33).

Furthermore, IRI can be mitigated by interfering with ferroptosis-related targets. The deubiquitinating enzyme USP22 can stabilize the sirtuin-1 (SIRT1) level to inhibit ferroptosis. USP22 overexpression can increase the SIRT1 protein level and decrease the p53 acetylation level, promoting SLC7A11 expression. Overall, this mechanism suppresses lipid peroxidation and attenuates ferroptosis-induced myocardial damage in IRI through SIRT1/P53/SLC7A11 axis (34). In the future, we can explore the clinical practice of USP22 on myocardial IRI to offer a novel diagnosis and therapeutic target for IRI patients.

### 3.3 Atherosclerosis

Atherosclerosis is a metabolic disease characterized by lipid metabolism and endothelial dysfunction. Atheromatous plaque formation is associated with iron deposition and peroxidation of lipids in vascular endothelial cells (35, 36). ACSL4 is up-regulated, while GPX4 is down-regulated in the coronary arteries of atherosclerosis patients (37). GPX4 controls the balance of reductive and oxidative states. GPX4 knockout can promote lipid peroxidation, leading to highly cytotoxic oxidation products for the cell and aggravating the atherosclerosis effect. Oppositely, GPX4 overexpression can alleviate atherosclerotic lesions of the aortic by inhibiting ferroptosis in ApoE^–/–^ mice (38). In addition, high level of uric acid has been shown to promote atherosclerotic plaque formation and inhibit the protein level of the NRF2/SLC7A11/GPX4 signaling pathway in ApoE^–/–^ mice (39). Fer-1 can inhibit iron deposition and lipid peroxidation in high-fat diet-fed ApoE^–/–^ mice by limiting SLC7A11 and GPX4 levels (36). Consequently, we can further explore the novel molecular targets that continue to involve in the atherosclerosis pathogenesis mechanism.

### 3.4 Chemotherapeutic drugs induced cardiotoxicity

Chemotherapeutic drugs induced cardiotoxicity remains an intractable issue for cancer patients, which is mostly associated with anthracycline drug (40). Doxorubicin (DOX), an anthracycline drug isolated from streptomyces, is frequently used to treat cancer patients (41). In DOX-induced cardiac injury mice, NRF2 induces HO-1 expression with an antioxidant effect, catalyzing hemoglobin degradation and promoting the free iron release, leading to ferroptosis and heart failure (42). Interestingly, protein arginine methyltransferase 4 (PRMT4) can modulate oxidative stress and autophagy, interacts with NRF2 to limit NRF2 nuclear translocation, and subsequently inhibits GPX4. A subsequent study confirms that PRMT4 overexpression raises ROS levels and intensifies DOX-induced myocardial dysfunction (43).

Additionally, mitochondria-dependent ferroptosis is involved in the pathology of DOX-induced cardiotoxicity. In the DOX-induced heart failure model, the GPX4 expression is downregulated, triggering lipid peroxidation of the DOX-Fe^2+^ complex and inducing mitochondria-dependent ferroptosis (44). Fer-1 and iron chelators can reduce DOX-induced cardiac damage by maintaining mitochondrial function (42). Therefore, these drugs can reduce DOX-induced cardiotoxicity by inhibiting the ferroptosis pathway, bringing good news to patients (42).

Tyrosine kinase inhibitors (TKIs) are also a class of anticancer agents for various cancers (45). Regorafenib, a molecule structurally related to sorafenib, is an effective xCT inhibitor, which can induce ferroptosis by decreasing cellular GSH (46, 47). In addition, lapatinib is usually used with the combined treatment of DOX to improve the anti-tumor efficacy. In H9C2 cells, lapatinib aggravates DOX-induced cell injury by decreasing GPX4 activity but increasing ACSL4 level (48). Together, these findings suggest that further in-depth research is required to study ferroptosis regulator genes as promising therapeutic targets in protecting TKIs-induced cardiotoxicity.

### 3.5 Heart failure

Heart failure is the terminal stage of various cardiovascular diseases (49). As mentioned above, iron homeostasis is crucial for maintaining cardiac function. Iron overload is closely related to heart failure and cardiomyopathy (50). For example, left ventricular diastolic function may be more sensitive to early markers of iron overload than systolic function (51). Furthermore, heart failure with preserved ejection fraction (HFpEF) patients has complex pathological processes, such as chronic inflammatory and oxidative stress stages. Elevated ROS level often promotes cardiomyocyte injury by increasing lipid peroxidation products, destroying the antioxidant mechanisms, and decreasing GSH levels. This progression implies an underlying connection between inflammation, ferroptosis, and HFpEF (51).

Of note, ferritin heavy chain (FTH), a significant component of ferritin, is down-regulated in transverse aortic constriction mice (49). SlC7A11 expression is decreased in FTH-deficient cardiomyocytes, while selectively, SlC7A11 overexpression increases the GSH level in cardiomyocytes (52). Puerarin, an antioxidant reagent, can alleviate heart failure by increasing FTH1 and GPX4 expression in H9C2 cells and aortic banding rats (53). Therefore, it can be shown that genes related to ferroptosis deserves for further exploration in heart failure.

### 3.6 Hypertension

Hypertension is a common comorbidity in HFpEF patients with high angiotensin II (Ang II) levels and myocardial fibrosis (51). Pathological cardiac remodeling mediated by hypertension leads to heart failure (54). The peptide hormone Elabela (ELA) is an endogenous ligand of the apelin receptor that can inhibit Ang II signal transduction, thus preventing pressure overloading. Intraperitoneal injection of ELA in Ang II-induced hypertensive mice model can inhibit interleukin-6/signal transducer and activator of transcription 3/GPX4 (IL-6/STAT3/GPX4) signaling, reducing the lipid peroxidation accumulation. Thus, ELA treatment in mice reduces myocardial fibrosis and cardiac injury with hypertensive heart failure (55). Similarly, it has been studied that the expression of GPX4 and GSH is decreased in hypertensive brain damage rat models (56). Moreover, SLC7A11 overexpression in mice alleviated Ang II-mediated cardiac fibrosis, hypertrophy, and dysfunction (57). At present, there is poorly existing basic research on the association between ferroptosis and hypertension, which is needed to deeper explore the underlying mechanisms, and provides new targets for the treatment of hypertension.

### 3.7 Other cardiovascular disease

Iron overload in diabetic patients increases the insulin resistance risk and aggravates cardiovascular complications through the Fenton reaction (50). Oxidative stress has become the main mechanism of diabetic cardiomyopathy (58). Heat shock factor 1 (HSF1) can resist oxidative stress response caused by ferroptosis-related lipid metabolism disorder. HSF1 overexpression alleviated palmitic acid-induced cell death and regulated the transcription of iron metabolism-related genes (FTH, TFRC, and FPN) to improve disturbed iron homeostasis (59). NRF2 is also a master regulator factor of antioxidant proteins in ferroptosis. Ferroptosis exacerbates diabetic cardiomyopathy by down-regulating the SLC7A11 expression through the AMPK/NRF2 pathway in the later stages of diabetes. Sulforaphane, an NRF2 inhibitor, prevents diabetes-induced oxidative stress and cardiac dysfunction by activating NRF2 (60). Likewise, AMPK/P38/NRF2 pathway is activated as an anti-oxidative stress mechanism during IRI in diabetic rats and is involved in the cardioprotective effect of resveratrol (61). Therefore, more investigation is needed to explore antioxidant drugs for treating diabetic cardiomyopathy.

Patients with severe sepsis often present with cardiac injury and dysfunction. Ferroptosis metabolic pathways such as mitochondrial autophagy and iron metabolism are involved in septic cardiomyopathy progression. Lipopolysaccharide (LPS) can increase ferritin and nuclear receptor coactivator 4 (NCOA4) expressions in H9C2 cells. NCOA4 increased the cytoplasmic Fe^2+^ and activated sideroflexin on the mitochondrial membrane to transport Fe^2+^ to mitochondria. This progress can lead to iron overload and elevate ROS in mitochondria, triggering lipid peroxidation and cardiomyocyte ferroptosis (62). However, FPN, as the only iron exporter in ferroptosis progression, is crucial for maintaining iron homeostasis. LPS can down-regulate FPN and up-regulate ferritin light chain (FTL) and FTH expressions, promoting iron deposition in myocardium-septic rats. Fer-1 and dexrazoridine can reduce cardiac inflammation and dysfunction and improve septic cardiomyopathy (62, 63).

## 4 Targeted therapy of ferroptosis in cardiovascular disease

Inhibiting ferroptosis-related targets has excellent therapeutic potential for treating and preventing heart disease, based on research into the pathogenesis of these conditions. In this section, we summarize various drugs that inhibit the ferroptosis pathway and their application in various models of heart diseases.

### 4.1 ROS inhibitors

Fer-1 can reduce ROS-induced cell damage and thus inhibit ferroptosis. Fer-1 is widely applied in various cardiovascular diseases (7). As described previously, Fer-1 has a protective effect on myocardial damage in DOX-induced and septic cardiomyopathy (42, 63). Fer-1 treatment reduced total creatine kinase release and neutrophil recruitment during heart transplantation (64).

Additionally, ferroptosis inhibitor liproxstatin-1 (LIP-1) has potentially cardioprotective properties. LIP-1 can reduce the myocardial infarction size by reducing voltage-dependent anion channel 1 (VDAC1) to maintain mitochondrial structure and function (65). MitoTEMPO, as a mitochondria-targeted superoxide scavenger, reduces lipid peroxides and thus significantly reduces cardiac dysfunction and mitochondrial damage (42). Hence, ferroptosis inhibitors are essential to treat cardiovascular disease. These drugs’ actual clinical development and utilization still need further exploration.

### 4.2 Iron chelators

Iron chelators can protect the myocardium from injury by regulating intracellular free iron levels. Dexrazoxane, a common iron chelator, easily passes through the cell membrane and chelates intracellular free iron. Dexrazoxane can act on high mobility group box 1 (HMGB1) protein to inhibit ferroptosis and reduce DOX-induced cardiotoxicity in rats (7, 66). Moreover, histochrome has better iron-chelating and antioxidant effects on alleviating myocardial IRI. Intravenous injection of histochrome in rats can inhibit ferroptosis by maintaining GSH level and GPX4 activity, thereby reducing infarct size and arrhythmia potential. The iron chelators deferiprone and deferoxamine can also inhibit IRI (67). However, further study is necessary to determine the effects of iron chelators on the body’s iron homeostasis.

### 4.3 Traditional Chinese medicine

Traditional Chinese medicine is a treasure trove of precious natural compounds with multiple targets and minor side effects (30). Some active ingredients of traditional Chinese medicine contain natural antioxidants and have regulatory effects on ferroptosis, such as artemisinin (68), curculigoside (69), curcumin (70), and glycyrrhiza (71). Several studies on alleviating cardiovascular disease with traditional Chinese medicine have progressed with in-depth research on ferroptosis (Table 1). For instance, baicalin can inhibit erastin-mediated GPX4 degradation and ACSL4 expression to enhance cell resistance to ferroptosis (72). Studies also showed that betulinic acid and ginsenoside Rd could inhibit oxidative stress markers and protect the heart from ischemia-reperfusion *via* NRF2/HO-1 signaling (73, 74). Besides, resveratrol can increase the GPX4 and FTH to reduce cardiac damage (75). Therefore, there is an urgent requirement to explore more traditional Chinese medicine with the ability to decrease lipid peroxidation and ROS to protect against cardiovascular disease with fewer side effects.

**TABLE 1:** Summary of traditional Chinese medicine in cardiovascular disease.

Drug	Mechanisms	Test in	Disease	References
Resveratrol	Increase the level of GPX4 and FTH, decrease the level of TFR1	Rat and H9C2 cells	Myocardial ischemia-reperfusion	(75)
Baicalin	Increase the level of GPX4, decrease the level of ACSL4, decrease the generation of ROS and Fe^2+^ deposition	Rat and H9C2 cells	Myocardial ischemia-reperfusion	(72)
Betulinic	Enhance the induction of nuclear NRF2 and HO-1 expression	Rat and H9C2 cells	Myocardial ischemia-reperfusion	(74)
Ginsenoside Rd	Increase the expression of nuclear NRF2 and HO-1	Rat	Myocardial ischemia-reperfusion	(73)
Cyanidin-3-glucoside	Increase the level of GPX4 and FTH1, decrease the level of TFR1	Rat and H9C2 cells	Myocardial ischemia-reperfusion	(76)
Hesperidin	Reduce non-heme iron deposited and lipid peroxidation	Mice	Iron-overload	(77)
Coumarin	Reduce lipid peroxidation	Mice	Iron-overload	(77)
Epigallocatechin-3-gallate	Increase the level of GPX4, decrease iron accumulation, inhibit excess ROS generation and oxidative stress	Mice and H9C2 cells	Dox-induced cardiomyopathy	(78)
Curcumin	Promote nucleus translocation of NRF2, increase the level of HO-1 and GPX4	Rabbit and H9C2 cells	Diabetic cardiomyopathy	(70)
Puerarin	Increase the expression of P-AMPK/T-AMPK; Increase the level of GPX4 and FTH1	Rat and H9C2 cells	Sepsis-induced myocardial injury; heart failure	(53, 79)
Shensong Yangxin	Increase the expression of TFR1 and FPN, decrease intracellular iron overload and ROS production	Rat and HL-1 cells	Syndrome-induced atrial fibrillation	(80)
Tanshinone	A coenzyme for NQO1, accept electrons from FAD to generate reduced tanshinone to reduce lipid ROS and ferroptosis	Mice	Myocardial ischemia-reperfusion	(81)

## 5 Discussion

**FIGURE 1:**
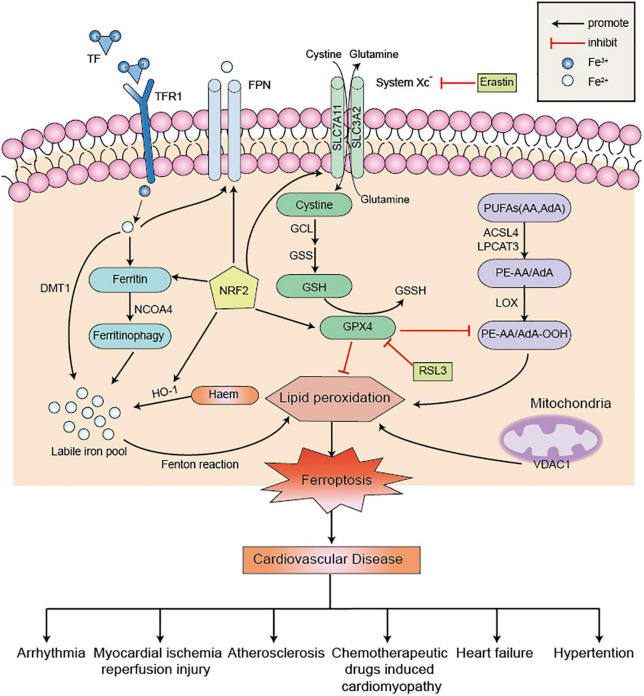
The regulatory metabolic pathways in ferroptosis. Ferroptosis is a form of cell death characterized by iron overload and lipid peroxidation. Excessive iron uptake or decreased iron excretion leads to intracellular iron overload, which promotes Fenton reaction. GPX4 is an essential regulator factor in the System Xc^–^-GSH-GPX4 axis system to blocking ferroptosis. Besides, PUFAs can be catalyzed by ACSL4 and LPCAT3 to form PE-AA and PE-AdA, which is further oxidized by LOX to promote the occurrence of lipid peroxidation. These three metabolic pathways promote ferroptosis and further aggravate cardiovascular disease. NRF2 also acts as an antioxidant factor to inhibit ferroptosis by operating its downstream factors. Erastin and RSL3 are common ferroptosis inducers to promote ferroptosis. PUFAs, polyunsaturated fatty acids; AA, arachidonic acid; AdA, adrenic acid; ACSL4, acyl-coa synthetase long chain 4; LPCAT3, lysophosphatidyl acyltransferase 3; LOX, lipoxygenase; System Xc^–^, cystine/glutamate antiporter; GSSH, glutathione disulfide; GSH, glutathione; TF, transferrin; TFR1, transferrin receptor 1; DMT1, divalent metal transporter 1; FPN, ferroportin; HO-1, heme oxygenase-1; NCOA4, nuclear receptor coactivator 4; NRF2, nuclear factor erythroid-2 related factor 2; RSL3, Ras-selective lethal small molecule 3; VDAC1, voltage-dependent anion channel 1; GPX4, glutathione peroxidase 4; PE, phosphatidylethanolamine.

As a non-apoptotic form of cell death, the underlying mechanism of ferroptosis is complex and intimately connected with other regulatory cell death signaling pathways (17). In-depth research on ferroptosis in cardiovascular disease has revealed that many signaling factors have been found to directly or indirectly regulate ferroptosis, thereby affecting iron metabolism and lipid peroxidation (Figure 1; 82). Recently, scientists are increasingly focusing on ferroptosis inhibitors to alleviate myocardial injury and cardiac dysfunction, which will provide insights into the molecular mechanisms of cardiomyocyte death after cardiac injury.

However, there are still many issues worth discussing: (1) The precise mechanisms involved in ferroptosis remain to be elucidated on cardiovascular disease. As mentioned above, studies exist on the experimental basis of ferroptosis and hypertension have been rare; (2) Further research is needed on the clinical application of ferroptosis and cardiovascular diseases, such as magnetic resonance imaging and serum-based biomarkers. By extension, we can explore predictive specific biomarkers of ferroptosis in cardiovascular disease, thereby providing a novel idea for early diagnosis and treatment of heart disease. (3) Traditional Chinese medicine has the advantage of its unique therapeutic effects, such as reduced toxicity and few side effects, in preventing and treating cardiovascular disease. There are more traditional Chinese medicine with anti-ferroptosis effect needs to be further investigated.

As a therapeutic target, ferroptosis has a good application prospect on cardiovascular disease (4). With the rapid development of molecular detection in the field of precision medicine, it need to be further explore more specific ferroptosis-related targets. Therefore, exploring the regulatory mechanism related to ferroptosis and actively promoting clinical verification is necessary to provide new treatment ideas and directions for clinical diagnosis and treatment of cardiovascular disease.

## Author contributions

HL and LL contributed to the literature review and manuscript drafting. Y-LX contributed to provide the funding. YX and XY were responsible for all the manuscript. All authors contributed to the article and approved the submitted version.
